# Artificial Intelligence to Detect Developmental Dysplasia of Hip: A Systematic Review

**DOI:** 10.1111/jpc.70172

**Published:** 2025-09-28

**Authors:** Suketu Bhavsar, Bhanu B. Gowda, Maulini Bhavsar, Sanjay Patole, Shripada Rao, Chandra Rath

**Affiliations:** ^1^ Neonatology, Perth Children's Hospital Nedlands Australia; ^2^ Neonatology, King Edward Memorial Hospital Subiaco Australia; ^3^ School of Medicine, University of Western Australia Nedlands Australia; ^4^ Neurology, Perth Children's Hospital Nedlands Australia; ^5^ KidsRehab WA, Perth Children's Hospital Nedlands Australia; ^6^ Edith Cowan University Joondalup Australia; ^7^ Canberra Hospital Garran Australia

**Keywords:** general paediatrics, neonatology, orthopaedics

## Abstract

**Aim:**

Deep learning (DL), a branch of artificial intelligence (AI), has been applied to diagnose developmental dysplasia of the hip (DDH) on pelvic radiographs and ultrasound (US) images. This technology can potentially assist in early screening, enable timely intervention and improve cost‐effectiveness. We conducted a systematic review to evaluate the diagnostic accuracy of the DL algorithm in detecting DDH.

**Methods:**

PubMed, Medline, EMBASE, EMCARE, the clinicaltrials.gov (clinical trial registry), IEEE Xplore and Cochrane Library databases were searched in October 2024. Prospective and retrospective cohort studies that included children (< 16 years) at risk of or suspected to have DDH and reported hip ultrasonography (US) or X‐ray images using AI were included. A review was conducted using the guidelines of the Cochrane Collaboration Diagnostic Test Accuracy Working Group. Risk of bias was assessed using the QUADAS‐2 tool.

**Results:**

Twenty‐three studies met inclusion criteria, with 15 (*n* = 8315) evaluating DDH on US images and eight (*n* = 7091) on pelvic radiographs. The area under the curve of the included studies ranged from 0.80 to 0.99 for pelvic radiographs and 0.90–0.99 for US images. Sensitivity and specificity for detecting DDH on radiographs ranged from 92.86% to 100% and 95.65% to 99.82%, respectively. For US images, sensitivity ranged from 86.54% to 100% and specificity from 62.5% to 100%.

**Conclusion:**

AI demonstrated comparable effectiveness to physicians in detecting DDH. However, limited evaluation on external datasets restricts its generalisability. Further research incorporating diverse datasets and real‐world applications is needed to assess its broader clinical impact on DDH diagnosis.

**Summary**

- What is known—what is new
  ○ Artificial intelligence in radiology is revolutionising diagnostic accuracy and efficiency by analysing medical images to assist in the detection, interpretation and diagnosis of various conditions.
  ○ Artificial intelligence‐assisted diagnosis of developmental dysplasia of the hip has the potential to achieve performance on par with that of experts.
  ○ Future research should focus on diverse datasets, image quality optimisation and automation to enhance artificial intelligence's clinical utility.

## 1 Introduction

Developmental dysplasia of the hip (DDH) affects 1 to 3% of infants and is characterised by a structural instability of the hip [1]. The severity of DDH ranges from mild dysplasia of the acetabular cavity to complete dislocation of the hip joint [2]. Delay in diagnosis of DDH can lead to early degenerative joint disease, excessive lumbar lordosis and chronic low‐back symptoms [3]. Hence, early detection and timely diagnosis are of paramount importance to improve outcomes and enhance cost effectiveness [3].

Early screening of DDH can be achieved by clinical examination, pelvic radiography and ultrasonography (US) of the hip. US can accurately examine all the components of an immature hip to detect dysplasia right from birth. It is portable, involves no radiation, and has a high sensitivity to detect even mild DDH [4]. However, the accuracy of measurements and classification depends on the standard plane, probe position and expertise. In addition, significant inter‐ and intraobserver variability has been reported [5, 6, 7]. Despite the limitations, US is one of the most important investigative tools for screening and diagnosis of DDH in neonates and infants.

The screening methodology for DDH may vary from targeted screening to extended screening to universal screening. Several countries have implemented universal DDH screening programmes using US [8]. A recent systematic review showed a trend toward lower prevalence of late DDH diagnosis with universal compared to selective screening [9]. However, universal screening results in excessive workload on the medical workforce. Irrespective of the methodology used, DDH screening involves obtaining massive number of images and reporting by competent specialists. Lack of a structured screening programme in the majority of the low‐ and middle‐income countries (LMIC) results in late diagnosis, morbidity and adds to the already existing economic difficulties [10]. In addition, LMICs have a shortage of specialised workforce, especially in smaller cities, where a major share of neonates is cared for. In this context, AI may have an immense role to play not only in moving tasks from man to machine but also in reducing the cost. Diagnosis of DDH with AI also has the prospect to be integrated into primary care and can potentially be cost effective. In a recent study of real‐time ‘Artificial intelligence (AI) decision support’ and a simplified portable US protocol enabled lightly trained primary care clinic staff to perform hip dysplasia screening with case detection and follow up rates similar to the experts [11]. It is also feasible to implement AI added DDH screening programme into primary care with a high patient satisfaction [12].

AI offers significant potential to automate manual tasks, ensure service consistency and improve patient care in radiology [13]. It is expected that AI methods complement physicians' expertise to improve diagnostic accuracy, reduce clinical workload and boost health economy. Several studies have evaluated the role of AI and deep learning (DL) in the diagnosis of DDH on pelvic radiographs or US [11, 14, 15, 16, 17, 18, 19]. A recent systematic review on this topic, which included 13 studies, reported a high efficacy of AI‐assisted imaging [20]. However, there have been many additional eligible publications for inclusion. Hence, we conducted this systematic review to evaluate the diagnostic accuracy of AI to identify DDH in infants.

## 2 Methods

This review was conducted using the guidelines of the Cochrane Collaboration Diagnostic Test Accuracy Working Group [21] and was reported using the Preferred Reporting Items for Systematic Reviews and Meta‐Analyses‐diagnostic test accuracy (PRISMA‐DTA) statement [22]. This systematic review was registered in the international prospective register of systematic reviews (PROSPERO ID: CRD42024543748). Due to the nature of this review, it was felt that ethical committee involvement was unnecessary.

### 2.1 Data Sources and Searches

PubMed, Medline, EMBASE, EMCARE, the clinicaltrials.gov (clinical trial registry), IEEE Xplore and Cochrane Library from inception until October 2024 were searched. Grey literature was searched on ‘Mednar’ database. PubMed was searched using the following terms: (‘hip dislocation, congenital’[MeSH Terms] OR (‘hip’[All Fields] AND ‘dislocation’[All Fields] AND ‘congenital’[All Fields]) OR ‘congenital hip dislocation’[All Fields] OR (‘developmental’[All Fields] AND ‘dysplasia’[All Fields] AND ‘hip’[All Fields]) OR ‘developmental dysplasia of hip’[All Fields] OR (‘hip dislocation, congenital’[MeSH Terms] OR (‘hip’[All Fields] AND ‘dislocation’[All Fields] AND ‘congenital’[All Fields]) OR ‘congenital hip dislocation’[All Fields] OR (‘congenital’[All Fields] AND ‘dislocation’[All Fields] AND ‘hip’[All Fields]) OR ‘congenital dislocation of hip’[All Fields]) OR (‘hip dislocation’[MeSH Terms] OR (‘hip’[All Fields] AND ‘dislocation’[All Fields]) OR ‘hip dislocation’[All Fields])) AND (‘artificial intelligence’[MeSH Terms] OR (‘artificial’[All Fields] AND ‘intelligence’[All Fields]) OR ‘artificial intelligence’[All Fields] OR (‘deep learning’[MeSH Terms] OR (‘deep’[All Fields] AND ‘learning’[All Fields]) OR ‘deep learning’[All Fields]) OR (‘neural networks, computer’[MeSH Terms] OR (‘neural’[All Fields] AND ‘networks’[All Fields] AND ‘computer’[All Fields]) OR ‘computer neural networks’[All Fields] OR (‘neural’[All Fields] AND ‘network’[All Fields]) OR ‘neural network’[All Fields]) OR (‘machine learning’[MeSH Terms] OR (‘machine’[All Fields] AND ‘learning’[All Fields]) OR ‘machine learning’[All Fields])). Similar terminologies were used while searching other databases. In addition to our electronic search strategy, we hand‐searched referenced lists of the relevant articles. No language or time restrictions were applied. The references from the database search were exported to ‘EndNote’ software (Version 20.6). Duplicate articles were removed, and the full texts of the eligible studies were obtained after reading the abstracts by two independent reviewers. The full‐text articles were read to assess their suitability for inclusion. In case of discrepancies, discussions were held with the third reviewer before reaching consensus.

### 2.2 Study Selection and Outcomes

We included prospective and retrospective studies that met the following criteria: (1) included children (< 16 years) at risk of or suspected or confirmed to have DDH; (2) reported hip US or X‐ray results using AI; (3) correlated artificial intelligence‐based results (AIBR) with the diagnosis made by the clinicians who are experts in interpreting such images. If the studies have reported outcomes of multiple models, the model with the best sensitivity was included in the systematic review.

The following types of studies were excluded: (a) Studies that have reported machine training data only, (b) Imaging done on operated hips, (c) Studies that enrolled ≤ 20 children, (d) Studies that enrolled a mixed population of infants, paediatric and adult patients and did not report the AIBR separately for the groups, (e) Studies that have compared AIBR with results reported by trainees/physicians with inadequate experience as defined by the studies and (f) Studies which have reported image quality and not DDH diagnosis data for analyses.

### 2.3 Data Extraction

Data was extracted using a standardised form. If required, the authors of original studies were contacted for additional information or clarifications. However, no one responded to our emails.

### 2.4 Assessment of ‘Risk of Bias’ and ‘Applicability Concerns’

The quality of included studies was assessed using the QUADAS‐2 tool (Quality Assessment of Diagnostic Accuracy Studies) [23] under the following key domains: patient selection, index test, reference standard and flow and timing. The RevMan V.5.2 software was used to generate tables and graphs of risk of bias assessment.

### 2.5 Data Synthesis

Data on true positives (TP), false positives (FP), true negatives (TN) and false negatives (FN) were collected from studies that categorised DDH results as either normal or abnormal. The TP, FP, TN and FN values were calculated using an online diagnostic test calculator (https://araw.mede.uic.edu/cgi‐bin/testcalc.pl) when studies reported only prevalence, sensitivity and specificity.

Given the significant heterogeneity in participant characteristics, AI tools used and timing of assessments, meta‐analysis was considered inappropriate. Hence, a narrative approach and diagrammatic representation to synthesise was undertaken.

In the absence of meta‐analyses, Certainty of evidence (COE) was graded as described by Murad et al. [24].

## 3 Results

**FIGURE 1:**
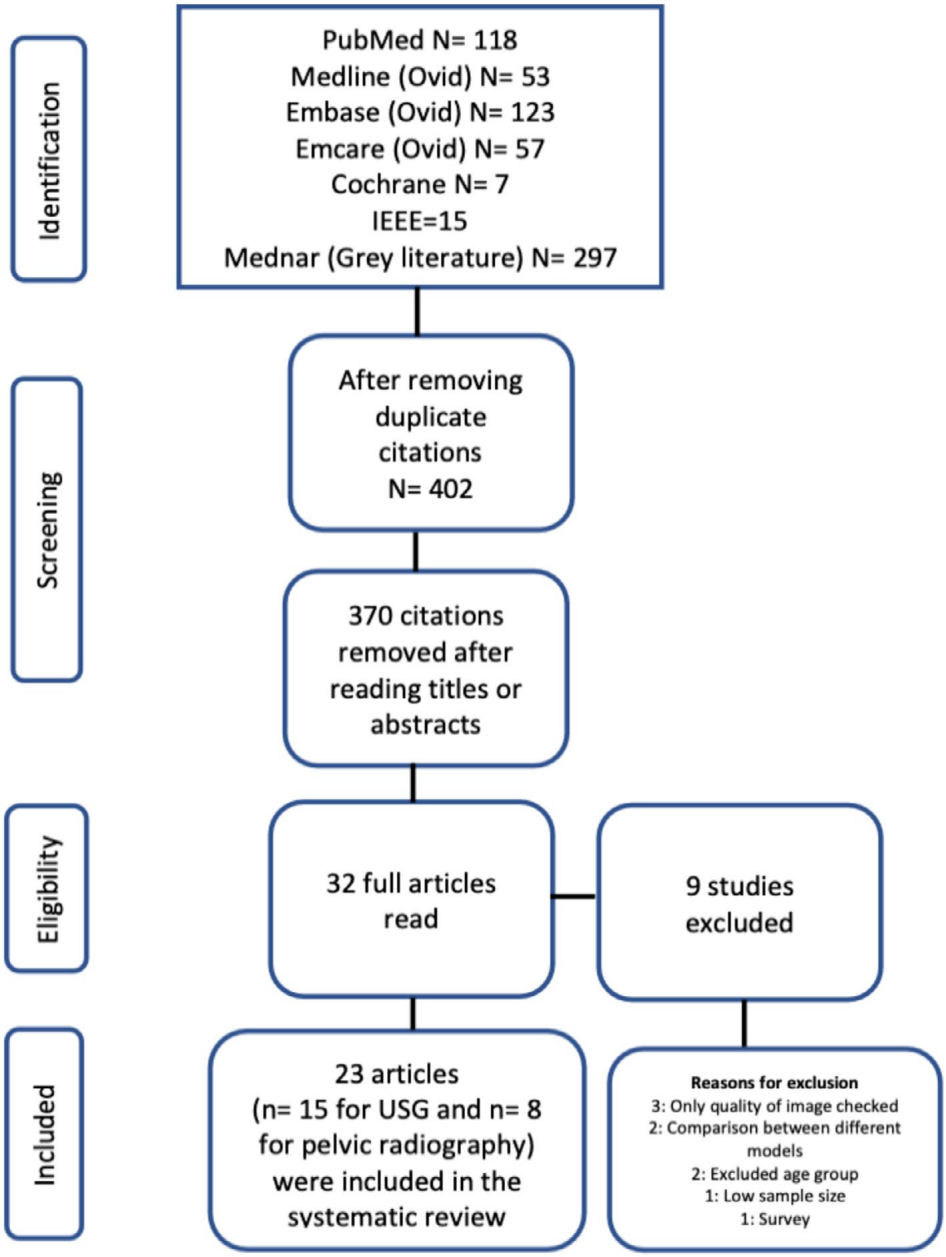
PRISMA flow chart for study selection.

The initial search retrieved 670 articles from the selected databases. Two hundred sixty‐eight duplicate articles were removed. Abstracts of the 402 citations were read, of which 32 were found to be potentially eligible. After reading the full articles of those 32 studies, 23 were found eligible for our systematic review and were included. The flow diagram for the study selection process is shown in Figure 1. For DL algorithm development, testing and to generate a ground truth for image labelling, 14 studies used consensus of a group of experts [15, 17, 18, 19, 25, 26, 27, 28, 29, 30, 31, 32, 33, 34], seven relied upon a single expert opinion [14, 16, 35, 36, 37, 38, 39] and two of the included studies used clinical diagnosis previously given by experts as a reference standard [40, 41].

### 3.1 Pelvic Radiograph

Eight studies reported on the diagnostic accuracy of AI/DL algorithms for the evaluation of DDH on pelvic radiographs [16, 25, 29, 30, 31, 32, 34, 39]. The included studies had a total sample size of 7091 images for AIBR testing. The individual study sample size ranged from 36 to 2276. The characteristics of the included studies are given in Table 1.

**TABLE 1:** Study characteristics: (A) pelvic radiograph and (B) ultrasonography.

Study ID, country, sample size for testing (images)	Age group	Machine learning model	Indices measured/objectives	Reference standard	Result	Conclusion
*(A) Pelvic radiograph*						
Sha 2023, China, 1086	4 months to 8 years	NA	Acetabular Index by Hilgenreiner method, IHDI classification. For severity. CEA angle.	Intermediate orthopaedicians (Attending surgeon)	AUC: 0.994, accuracy: 99.72%, Sn: 100.00%, and Sp: 99.59% Mean time to diagnosis for AI was 10.66 ± 0.70 s vs. 156.62 ± 7.40 s for the clinicians. ICC: 0.953	The software can provide expert‐like analysis of pelvic radiographs and obtain the radiographic diagnosis of paediatric DDH with great consistency and efficiency.
Den 2023, Japan, 47	< 12 months	YOLOv5 Four different models (YOLOv5s, YOLOv5m, YOLOv5l, YOLOv5x)	IHDI classification DDH vs. Normal Hip	Paediatric hip specialist	YOLOv5L achieved best Sn 0.94 (95% CI 0.73–1.00) and Sp 0.96 (95% CI 0.89–0.99)	YOLOv5 DL model provides good diagnostic performance for DDH and it is a useful diagnostic assistant tool.
Wu 2023, China, 1000	10 days to 10 years, four subdivisions, < 6 , 6–12, 12–24, > 24 m	Faster R‐CNN DDH network (FRCNN)	Acetabular Index by Hilgenreiner method. Hip joint graded according to Tönnis criteria	13 Expert clinicians (paediatric orthopaedics and radiologists) committee consensus. 10 clinicians −8 years' experience, two clinicians with 15 years' experience, one clinician with 25 years' experience	Machine vs. reference standard 95% LOA by Bland–Altman plot was −4.02° to 3.45° (bias = −0.27°, *p* < 0.05)	The acetabular index measured by the system within all age groups showed good credibility according to Bland Altman principle. The accuracy of the system is closer to that of senior clinicians.
Fraiwan 2022, Jordan, 36	3–7 months	DarkNet‐53 DenseNet‐201 EfficientNet‐b0 GoogLeNet Inceptionv3 Inception‐ResNet Mobile‐Netv2 ResNet‐18, ResNet‐50, ResNet‐101 ShuffleNet SqueezeNet Xception	DDH vs. Normal Hip. (Direct result output)	Three specialist consensuses	DarkNet 53 achieved highest mean DDH detection accuracy: 96.3%, F1 score: 95%, precision: 90.6%, Sn: 100%, Sp 94.3%	Automated method appears to be a highly accurate for DDH screening and diagnosis
Park 2021, Korea, 513	< 12 months	DL algorithm	DDH vs. Normal hip	Two paediatric radiologist (7 and 13 years of experience) consensuses	Sn (94%), Sp (98.9%), PPV (90.4%) and NPV (99.4%), AUROC 0.988 and AUPRC of 0.973. There was no significant difference in diagnosis of DDH by DL algorithm and experienced radiologists (*p* = 0.180). DL algorithm showed higher performance in DDH diagnosis compared to radiologist without experience (*p* < 0.001)	The DL algorithm provided an accurate diagnosis of DDH on hip x‐rays, which was comparable to the diagnosis by an experienced radiologist
Xu 2021, China, 133	6 months to 3 years	Mask R‐CNN, HRNet, ResNet	Centre edge angle, acetabular index, Shenton's line, lateral edge of acetabular, Sourcil of the acetabulum	Orthopaedic senior surgeons with 10 years' experience	Accuracies: 0.86 to 0.95. Sn for Tönnis and IHDI classification: 0.84 to 0.96. The AI system was close to the intermediate surgeon, more sensitive than the junior surgeon, and has high Sp. ICC: excellent agreement between all surgeons and AI (ICC > 0.75). Average time consumption: AI: 1.21 s, SS: 150.36 ± 26.24, IS: 200.71 ± 25.71, JS: 172.92 ± 20.58 s (AI vs. surgeons: *p* < 0.001)	AI‐aided diagnostic system can quickly and automatically measure important parameters and improve quality of clinical diagnosis.
Liu 2020, China, 2000	1 month to 6 years	PN‐Unet	Landmark detection, Acetabular index and DDH diagnosis	Orthopaedic surgeons	Landmark detection: PN‐Unet successfully detected 90% with precision range of 2.5 mm. Acetabular index: Accuracy was 90.10% for right hip and 89.80% for left hip. DDH diagnosis: Recall (Sn) 92.86%, precision 96.02%, F1 score 94.68%. AI took average < 1 s to diagnose heavily dislocated hip, doctors with CAD took average 60 s and doctors without CAD took 200 s.	PN‐Unet exhibits excellent performance in DDH diagnosis
Zhang 2020, China, 2276	0 to 10 years. Subgroups: 0–2 years and > 2 years	FR‐DDH network	IHDI and Tönnis classification Acetabular index	Expert committee consensus (two paediatric orthopaedicians, two paediatric radiologists)	DL system achieved overall AUC 0.975, accuracy 99%, Sn 95.5% and Sp 99.5%. 0–2 years: AUROC 0.974, Accuracy 98.9%, Sn 95.3%, Sp 99.5%. Infants < 6 m: AUROC 0.952, Accuracy 98.9%, Sn 90.5%, Sp 100%. > 2 years: Accuracy 100%. Mean time to process one AP x‐ray by DL was1 second vs. 30 s by surgeons	The DL system was highly consistent and was more convenient and effective for diagnosing DDH compared to clinicians.
*(B) Ultrasonography*						
Li 2024, China, 1500	30–90 days	ResNext‐FPN‐50 (feature pyramid network) and contrastive learning	Alpha and beta angle (Graf method)	Six orthopaedic surgeons with extensive experience	False positive ratio of the proposed method was decreased by 39.4%, manual operators had higher false negative ratio 9.4% than the algorithm (5%).	Semi‐supervised DL method following Graf's principle, can identify landmark on images more accurately and improve the efficiency of diagnosing hip dysplasia in infants.
Kinugasa 2023, Japan, 214	Up to 6 months	SqueezeNet, MobileNet, EfficientNet	Graf method	Two paediatric orthopaedics experts	Efficient Net model: Accuracy, recall (sensitivity), F1 score and precision 1.0. SqueezeNet and MobileNet achieved accuracy of 0.99 and 0.95 respectively. The AUC for all the models was 0.99	USS imaging with DL can assess DDH with high accuracy
Atalar 2023, Turkey, 243	Infants	VGG‐16, Resnet‐101, GoogLeNet, Mobilenet v2	Graf methods, Abnormal hip, dysplastic hip, Probe malpositioning	Orthopaedic specialist (single)	VGG‐16 model achieved the best results: 93% accuracy, 93.5% Sn, 96.7% Sp, 92.3% precision, 92.6% F1 score and 0.99 AUC	DL method successfully evaluated USS images of dysplastic hip, healthy hip and probe malpositioning
Chen 2023, Taiwan, 434	Infants 0–6 months	UNet++	Graf method, Alpha < 60, alpha angle < 50 and beta angle < 77	Review board of three senior paediatric orthopaedicians consensus	Unet++ model demonstrate precise key point location. Model achieved key points detection of 95% with error distance < 2 mm. Linear r for alpha angle = 0.89 (*p* < 0.001) and 0.68 (*p* < 0.001) for Beta angle AUC of 0.937 for classifying alpha < 60^°^, 0.974 for alpha angle < 50^°^ and 0.974 for classifying beta angle > 77^°^. On average expert agreed to 97.6% of model inference images.	DL model can be an efficient tool for assisting DDH diagnosis in clinical settings.
Huang 2023, China, 369	0–6 months	DDHnet	Graf method, Alpha angle, Beta angle, FHC, PFD	Senior expert (10 years' experience) in paediatric USS	Interclass r of abnormal hips, Alpha angle: 0.96 (95% CI 0.93–0.97), Beta angle 0.97 (95% CI 0.95–0.98), FHC 0.98 (95% CI 0.96–0.99), PFD 0.94 (95% CI 0.90–0.96), AI vs. Reference standard: Sn 90.56%, Sp 100%, Accuracy 98.64%, PPV 100%, NPV 98.44%, Kohen kappa 0.943 (0.894–0.992) and agreement was 98.64%, DDHnet took only 1.1 s to diagnose compared to expert who required 41.4 s	The proposed DDHnet demonstrates fast and highly accurate DDH diagnosis.
XU 2023, China, 220	14 days to 6 months	AI model	Alpha angle, Beta angle, FHC	Two chief physicians	AI model automatically identify standard coronal plane of hip joint USS with accuracy of 96.70% and had good agreement with chief ultrasound physicians (Cohen's kappa = 0.925, *p* = 0.007). Interclass *r* between AI model and chief physician for alpha angle 0.814 (95% CI 0.764, 0.854), beta angle 0.730 (95% CI 0.661, 0.786), FHC 0.953 (95% CI 0.939, 0.963) (*p* < 0.001)	AI model could be used to automatically identify the standard coronal section of hip USS and measure relevant parameters effectively, hence assisting ultrasound screening of DDH in infants
Ghasseminia 2022 (A), Canada, 240	4–267 days	MEDO‐Hip (UNet like CNN model)	Graf methods, Alpha angle, FHC, DDH diagnosis	Three DDH specialists	AI achieved moderate agreement with clinical diagnosis (kappa 0.47 to 0.49), AI more consistent between 2D US images and sweep images (ICC 0.90 vs. 0.87 for alpha angle and ICC 0.91 vs. 0.91 for hip coverage) against widely accepted standard (measurements from three DDH specialists). AI reliability deteriorated more than human readers for poorest quality images	AI performance was intermediate between expert readers and less‐experienced human readers on these challenging data set
He 2022, China, 216	0–150 days	3D ultrasound + AI	Graph method, Alpha angle	Trained doctor	The measured α value of the optimal section obtained by 3D US showed good agreement with the measured α value of the standard Graf section. Sensitivity 88.8%, Specificity 89.3%, accuracy 93.8%, AUC 0.938.	The AI and 3D US‐based automatic evaluation technology for section selection and inspection for DDH showed good agreement with the Graf method based on standard sections.
Lee 2021, Korea, 921	Infants	Mask R‐CNN, AI	Graf method, Alpha angle, Beta angle, Check angle	One radiologist and one orthopaedic surgeon expert (> 10 years' experience)	Excellent agreement in the Interclass r value between AI and experts for alpha angle (Interclass *r* = 0.764) (0.699–0.815) and a good agreement in beta angle (Interclass *r* = 0.743) (0.689–0.788). Moderate agreement was seen in check angle measurement (80.9%, Cohen's k = 0.525)	DL model performance was comparable to human experts.
Gong 2021, China, 758	0–6 months	TML‐DERM	Statistics and Texture of hip joint image	Two experienced sonologist consensus	Accuracy: 85.89%, Sn: 86.54%, Sp:85.23%, PPV: 85.43%, NPV: 86.38%, AUROC: 0.8919	Experimental results show that the proposed TML‐DERM outperforms all the comparative algorithms on a real‐world data set of infantile DDH
Hu 2022, China, 247	0–6 months	ResNet FPN‐101 based multitask learning network algorithm	Graf method, Assessment of dysplasia metrics, alpha angle and beta angle	Labelled by one senior doctor and reviewed by Expert senior doctors	New algorithm performed better than manual plots by doctors. Mean accuracy of this model for alpha angle was 93.456% and for beta angle was 84.615%. The Pearson r of the alpha and beta angles are 0.88 and 0.89. Mean absolute difference (SD) for alpha and beta angle was 2.221 (2.007) and 2.899 (2.283) respectively.	Proposed algorithm can accurately and robustly realise the automatic evaluation of DDH
Sezer 2020, Turkey, 203	0–6 months	Deep CNN	Graf method, CNN extracted shape and texture characteristics of US images (Alpha and beta angles not measured)	Single expert with > 10 years' experience	Accuracy rate of proposed CNN on original data set was 92.29%, which increased to 97.70% on augmented data set. On original data set proposed CNN has success rate of 96.1% for classification of normal hip, 84% for type IIa and IIb and 86.79% for type IIc and D	Deep CNN based automatic classification of DDH, classifies neonatal dysplastic hip with high accuracy. Accuracy of CNN improves with data augmentation.
Ghasseminia 2022 (B), Canada, 2492	4–267 days	MEDO‐Hip (UNet like CNN model)	Alpha angle, FHC	Clinical diagnosis	AI correctly classified 90% of dysplastic hips and 86% of other hips overall with Sn of 90% for dysplasia. Agreement between AI and clinical diagnosis was very high (kappa *r* = 0.79). Comparing AI analysis of 3DUS vs. reference standard 0.90 Sn, 0.86 Sp, 99.2% NPV, 32% PPV. The Interclass r for AI vs. clinical alpha angles were 0.56 (95% CI 0.40–0.66, *p* < 0.001) across the two centres.	Automated AI analysis of 3D US images had high diagnostic accuracy for classification of infant hips as normal or dysplastic compared to reference‐standard clinical diagnosis made using 2DUS.
Oelen 2022, Switzerland, NA	Newborn	VGG 16, RESNET, U‐NET, PSP‐NET	Alpha angle, Landmark detection and line detection	Physicians	The RMSE between landmark detection and ground truth was 3.9° for alpha. The RMSE between physician and ground truth was found to be 7.1°.	The accuracy of physicians in their daily routine is inferior to DL‐based algorithms.
Quader 2017, Canada, 258	Infants	Automatic methods	Alpha angle, Beta angle, FHC, Graf classification	Radiologist	Discrepancies between AI vs. manual measurement of alpha angle was modest and statistically significant (mean 1.8° SD 4.7°, *p* < 0.01), where those for Beta angle and FHC were larger and statistically significant (beta angle mean 8.9°, SD 6.4°, *p* < 0.01; FHC mean −7.4%, SD 6.9% *p* < 0.01). DDH classification: 91% agreement. Time taken nearly 1 s.	The proposed method produced excellent agreement with radiologists in scan adequacy classification and significantly reduced measurement variability in the dysplasia metrics.

Abbreviations: AA, acetabular angle; AI, artificial intelligence; AP, anteroposterior; AUPRC, area under the precision‐recall curve; AUROC, area under receiver operating characteristic curve; CAD, computer aided diagnosis; CEA, central edge angle; CNN, convolutional neural network; DDH, developmental dysplasia of the hip; DL, deep learning; DNMSVM, deep neural mapping SVM; ExRM, exclusivity regularised machine; FHC, femoral head coverage; ICC, intraclass correlation coefficient; IHDI, International Hip Dysplasia Institute; IS, intermediate surgeons; JS, junior surgeons; LOA, limits of agreement; MAE, mean absolute error; NA, not available; NPV, negative predictive value; PFD, pubo‐femoral distance; PPV, positive predictive value; *r*, correlation coefficient; RF, random forest; RMSE, root mean square error; SD, standard deviation; Sn, sensitivity; Sp, specificity; SS, senior surgeons; SVM, support vector machine; TML‐DERM, two staged meta‐learning based deep exclusivity regularised machine; USS, ultrasound scan.

**FIGURE 2:**
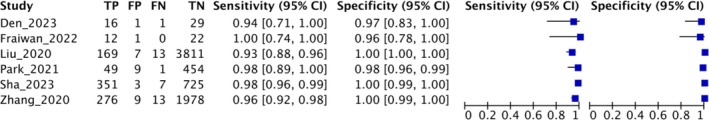
Reported or calculated sensitivity and specificity (95% CI) of AI‐based pelvic radiography. CI, confidence interval; FN, false negative; FP, false positive; TN, true negative; TP, true positive.

The studies were conducted on an age group of 0–10 years. Pelvic radiograph reported by an experienced orthopaedic surgeon or paediatric radiologist was used as a reference. Measurement objectives used to evaluate the severity of hip dysplasia on pelvic radiographs were acetabular index, acetabular angle, centre edge angle, Shenton's line, lateral edge of acetabulum, sourcil of acetabulum as per international hip dysplasia institute (IHDI) and Tönnis classification standard. The AUC of the DL algorithm for hip dysplasia on pelvic radiograph in the included studies ranged from 0.88 to 0.99. The reported/calculated accuracy ranged from 86% to 100%. The studies evaluating the performance of the DL model on X‐ray hip images reported a sensitivity ranging from 92.86% to 100% and a specificity ranging from 95.65% to 99.82% (Figure 2). Positive likelihood ratio (PLR) and negative likelihood ratio (NLR) were reported/calculated for six studies [16, 25, 29, 30, 34, 39]. The reported PLR and NLR ranged from 23 to 506 and 0 to 0.07, respectively. The reported/calculated DOR ranged from 464 to infinite. Two studies reported correlation between physicians and the AI model and found it to be excellent (Interclass correlation coefficient [ICC] > 0.75) [32, 39]. Four of the included studies reported time taken by the DL algorithm for image evaluation in comparison to doctors [29, 32, 34, 39]. The DL model took a mean time ranging from < 1 s to 10.66 s, while doctors took a mean of 30–160 s for image evaluation.

### 3.2 Ultrasonography

Fifteen studies reported the role of AI/DL algorithm for diagnosis of DDH on US images [14, 15, 17, 18, 19, 26, 27, 28, 33, 35, 36, 37, 38, 40, 41]. The included studies used a total of 8315 US images for AIBR testing. The individual study sample size ranged from 203 to 2492.

Studies evaluating AIBR included children aged from 0 to 12 months. US images were evaluated with alpha angle, beta angle, check angle, femoral head coverage (FHC), pubo‐femoral distance. The majority of the studies measured alpha angle to determine hip dysplasia by Graf classification [14, 15, 17, 18, 19, 27, 28, 33, 35, 36, 37, 40, 41]. Six studies also measured beta angle to determine the severity of hip dysplasia [15, 17, 27, 33, 36, 37]. In addition to alpha and beta angle, five studies measured FHC to diagnose hip dysplasia [33, 36, 37, 40, 41]. Two studies used the shape and texture of the hip joint in USS images to determine hip dysplasia [26, 38].

**FIGURE 3:**
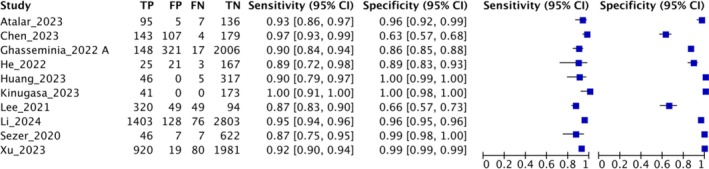
Reported or calculated sensitivity and specificity (95% CI) of AI‐based ultrasonography. CI, confidence interval; FN, false negative; FP, false positive; TN, true negative; TP, true positive.

The AUC of the DL algorithm for DDH ranged from 0.90 to 0.99 among the included studies. Studies evaluating performance on US hip images reported sensitivity of 86.54%–100% and specificity of 62.5%–100% in detecting DDH (Figure 3). PLR and NLR were reported/calculated for 10 studies [14, 15, 18, 27, 28, 33, 35, 36, 38, 41]. The reported PLR and NLR ranged from 2.59 to infinite and 0–0.2, respectively. The reported/calculated DOR ranged from 12.5 to infinite.

The accuracy of the AI/DL algorithm to measure alpha and beta angles on US images was 81%–100%. Six studies have reported agreement between the AI/DL algorithm and experts to measure the alpha angle, beta angle and to identify hip dysplasia on US images. The reported agreement ranges from 0.5 to 0.94 [27, 33, 36, 37, 40, 41]. Six studies reported ICC between AI and experts for alpha angle measurement. Five out of six studies reported excellent correlation (ICC = 0.76–0.96) [15, 27, 33, 36, 41] while one study reported fair correlation (ICC = 0.56) [40]. One study reported a mean machine time of < 1 s compared to 41.4 s by the experts to evaluate the US images [36].

A meta‐analysis was not performed due to substantial heterogeneity across the included studies. In addition to the use of various AI tools, there were notable differences in the age at which imaging was conducted and in the specific indices measured—such as the alpha angle, beta angle, check angle, acetabular index, acetabular angle, centre‐edge angle and Shenton's line. Given this variability, conducting a meta‐analysis was deemed inappropriate. Since meta‐analysis was not undertaken, publication bias was not assessed. The COE was deemed low, in view of significant heterogeneity and small sample size in few of the included trials (Table 2).

**TABLE 2:** GRADE evaluation of AI‐based ultrasonography and pelvic radiography in diagnosing developmental dysplasia of the hip.

Outcome	No. of studies (No. of patients)	Study design	Factors that may decrease certainty of evidence					Test accuracy CoE
			Risk of bias	Indirectness	Inconsistency	Imprecision	Publication bias	
Ultrasonography	15 studies patients (8315)	Cross‐sectional (cohort type accuracy study)	Not serious	Not serious	Serious^a^	Not serious	Publication bias strongly suspected^b^	⊕⊕○○ Low^a^, ^b^
Pelvic radiograph	Eight studies patients (7091)	Cross‐sectional (cohort type accuracy study)	Not serious	Not serious	Serious^a^	Serious^b^	Publication bias strongly suspected^c^	⊕⊕○○ Low^a^, ^c^

^a^ Substantial heterogeneity in terms of artificial intelligence tool used.

^b^ Few trials had small sample sizes and wide confidence intervals.

^c^ < 10 studies.

### 3.3 Risk of Bias

**FIGURE 4:**
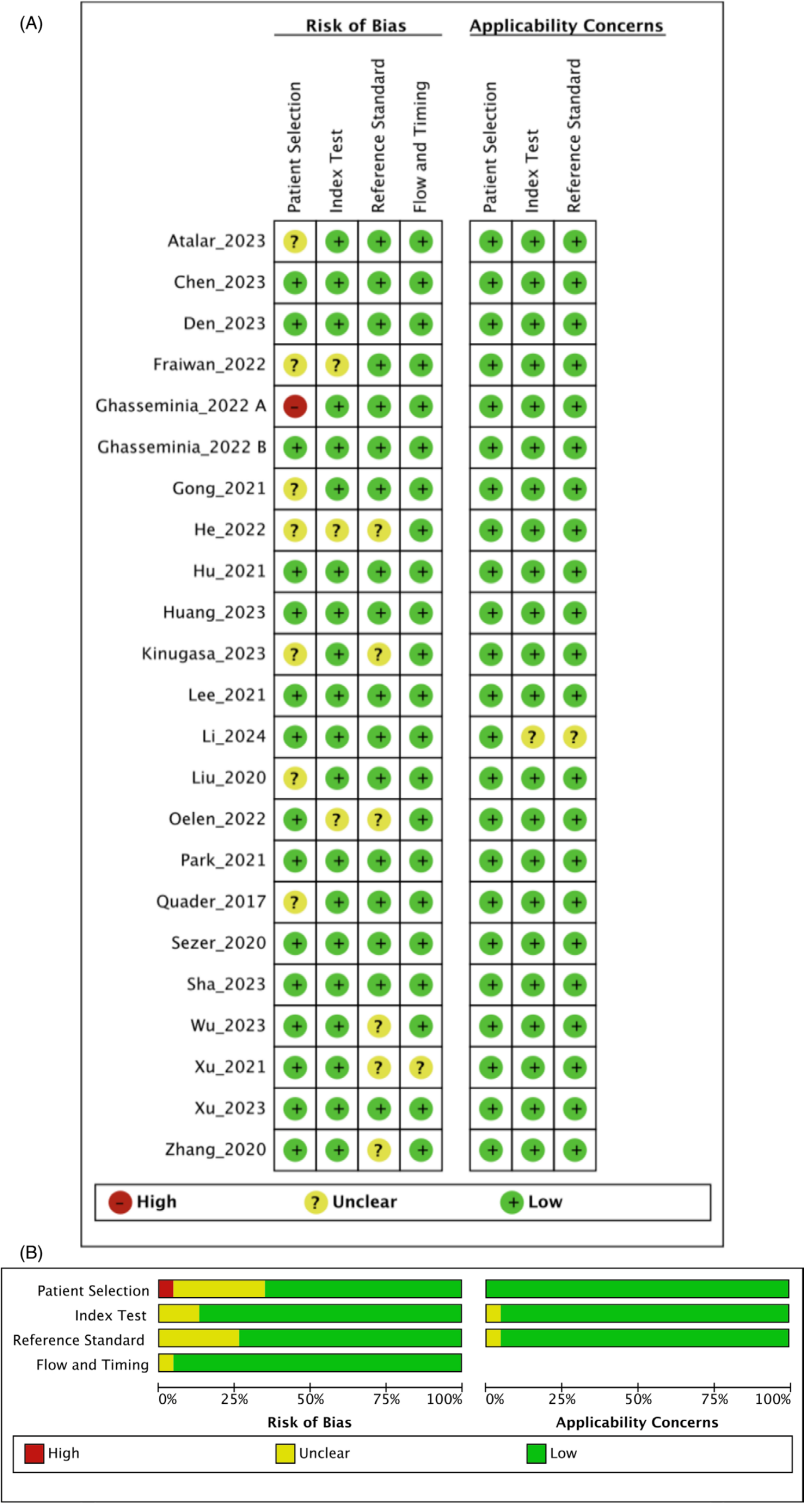
Summary of the risk of bias and applicability concerns.

Majority of the included studies had low risk of bias (Figure 4A,B). We did not identify any applicability concerns. 40% of the included studies had either unclear or high risk of bias in the domain of patient selection and 20% of the studies had unclear bias in the domain of reference standard.

## 4 Discussion

Our systematic review that included 23 studies showed that AI can detect DDH with very high levels of sensitivity, specificity and the results correlate well with the reference standard.

Diagnosis of DDH with the help of AI is not without problems. Though AI may be a useful tool to diagnose DDH, diagnostic accuracy on a poor‐quality image needs further deliberation [42]. It is important to obtain good‐quality images as AI works best if the image standard is superior [43]. In a trial environment, the researchers tend to choose good images where labrum and femoral heads are clearly identified. Hence, application of these AI technologies in real‐world environment might bring the diagnostic accuracy down. Researchers have used various technologies to mitigate this issue. A study suggested saving US sweep for analysis later so that AI can choose the best possible image to diagnose DDH [41]. Such an approach might alleviate the difficulty of obtaining a good‐quality image in a crying and unsettled infant. In addition, this can be undemanding for a less experienced operator. Another study that used extracted discriminative shape and texture features based on a combination of neural networks rather than angle measurements has reported a reasonable success rate [38]. Interrater reliability is an issue for alpha angle measurement in two‐dimensional US images [37]. Three‐dimensional images provide compendious visualisation of the hip joint and are easy to obtain by a novice operator. In a study, three‐dimensional imaging significantly improved interrater reliability in novice readers [44]. In a recent study, AI analysis of three‐dimensional US images correlated well to human expert diagnosis of DDH from concurrent conventional 2D ultrasound of the same hips [40]. Similarly, the acetabular index plays an important role in diagnosing DDH on pelvic radiographs. The acetabular index changes with age [45] and consistency of measurement is worse in < 6 months of age than in the older age group [31]. In addition, the DL methods may not be able to automatically identify the rotation and tilt of the image, nor can they automatically correct the errors caused by the tilt rotation. In this context, application of AI for detection of DDH needs further refinement in specific indicators.

We included 10 additional studies compared to the previous systematic review [20]. Due to significant heterogeneity in the AI tools used, we decided against conducting a meta‐analysis. Unlike the previous review, we excluded studies focused on using AI to assess image quality. Instead, we only included studies that reported the use of AI specifically for diagnosing DDH.

The strengths of our systematic review include the use of robust methodology, assessment of risk of bias and a comprehensive literature search. Our systematic review has several limitations. The degree of training of DL models was not considered. Second, the occurrence of hip dysplasia is multifactorial, such as gender, race, family history and breech presentation, which may influence the output of the DL algorithm. This systematic review did not take these confounding and demographic factors into account. There is heterogeneity in terms of AI models used, and we are unable to recommend any one model for evaluating hip dysplasia either on US or pelvic radiograph images. We also acknowledge heterogeneity in participant characteristics, method of reporting of diagnostic accuracy and lack of data suitable for pooling. It is a well‐known fact that heterogeneity is inherent to diagnostic accuracy studies in view of the nonrandomised design.

## 5 Conclusion

This systematic review provides an overview of the available evidence on AI application on pelvic radiographs and USS images for diagnosis of DDH in children. AI‐assisted diagnosis of DDH has the potential to match expert‐level performance. Future research should focus on extensive training using diverse datasets and various models, along with advancements in technology that could address current challenges and enhance the generalisability of DL models.

## Author Contributions

All authors contributed to the study conception and design. Material preparation, data collection and analysis were performed by Suketu Bhavsar, Chandra Rath, Bhanu Gowda B. and Maulini Bhavsar. The first draft of the manuscript was written by Suketu Bhavsar and Chandra Rath. All authors commented on previous versions of the manuscript. All authors read and approved the final manuscript.

## Ethics Statement

The authors have nothing to report.

## Conflicts of Interest

The authors declare no conflicts of interest.
